# Trends in the practice environment of Chinese healthcare professionals from 2008 to 2023: an age period cohort analysis

**DOI:** 10.1186/s12960-024-00954-5

**Published:** 2024-11-13

**Authors:** Liangquan Lin, Yi Che, Jiaxin Zhou, Yixin Gui, Xinqing Zhang

**Affiliations:** 1 School of Marxism School of Humanities and Social Sciences, Chinese Academy of Medical Sciences & Peking Union Medical College, #9 Dongdan, 3nd Alley, Beijing, 100730 China; 2 The Affiliated Hospital of Changchun University of Chinese Medicine, Changchun, 130021 China

**Keywords:** Healthcare professionals, Age-period-cohort analysis, Practice environment, Perception, Trend

## Abstract

**Background:**

Healthcare practice environment plays a vital role in evaluation and the development of health sector in China. However, there are few comprehensive reviews and studies focusing on its state and changing trends. This study aimed to examine the dynamic trends in Chinese healthcare professionals’ perceptions of their practice environment from 2008 to 2023 using age period cohort (APC) analysis.

**Methods:**

Four national cross-sectional surveys of healthcare professionals were conducted in 2008, 2013, 2018, and 2023. APC analysis was performed to distinguish effects of age, period and cohort. Covariates like gender, department, job satisfaction, and doctor–patient relationships were also analyzed.

**Results:**

Between 2008 and 2023, healthcare professionals' perceptions of their practice environment first declined and then improved. Those aged 28–38 during 2013–2018 and born between 1978 and 1988 had the most negative perceptions. After 2018, perceptions improved, peaking in 2023. Those under 23 and over 43 exhibited larger age effects. Birth cohorts after 1993 also had more positive effects. Controlling for covariates attenuated APC effects. Females, those in obstetrics and emergency medicine, nurses, technicians, and administrators perceived better environments. Higher job satisfaction and doctor–patient relationship harmony are also associated with more positive perceptions. Income matching efforts and perceptions of promotion fairness had positive impacts, while increasing severity of physical fatigue and psychological anxiety negatively influenced perceptions of the practice environment.

**Conclusions:**

The APC analysis provided nuanced insights into evolving practitioner perceptions amid healthcare reforms in China. Tailored policies focused on career stage and generation are needed to address disruptions and sustain improvements. Monitoring feedback on reforms and changes is essential for optimizing the practice environment over time.

**Supplementary Information:**

The online version contains supplementary material available at 10.1186/s12960-024-00954-5.

## Introduction

The practice environment is defined as “the characteristics of an organization that either facilitate or restrain the professional nursing practice environment [1]. A positive practice environment leads to higher work efficiency, and better treatment outcomes, and reduces burnout, sick leave, and turnover rates [2, 3]. A study in the United States found that after improving the nursing practice environment in rural and small hospitals in Texas, nurse retention rates, nurse staffing, and quality of care all improved [3]. Additionally, the change of healthcare professionals' perception of the practice environment also confirms the effect of healthcare reforms [4]. Unfair salary and promotion, tense doctor–patient relationships (DPR) and workplace violence, high work pressure and night shifts, physical fatigue and psychological anxiety are factors significantly affecting the perception of healthcare practice environment and are indicators of concern in healthcare reform [5]. Therefore, conducting a systematic examination of the shifts in the perceptions of practice environment of healthcare professionals before and after the comprehensive health reform is essential for evaluating the reform outcomes effectively. Healthcare professionals' perceptions of reforms are highly significant. Studies interviewing healthcare professionals in Turkey, southern Australia, and New Zealand have revealed the facilitating role of their perspectives in the reform process. It was also found that the government's failure to involve healthcare workers in the formulation and implementation of health policies has left them feeling vulnerable [6–8].

In 2009, China initiated a comprehensive health reform. Within the 10 years, it had almost established a basic health care system covering all the population [9]. The healthcare reform has made commendable progress through the implementation of a series of policies that optimize the practice environment for healthcare professionals. China has a tiered healthcare delivery system consisting of primary, secondary and tertiary institutions, while tertiary healthcare is provided by large specialized hospitals and general hospitals located in counties and cities [10]. Public hospitals dominate China's medical service system, taking up 89% of hospital beds and 92% of hospital admissions [11]. Healthcare financing relies heavily on social health insurance schemes covering over 95% of the population [12]. By the end of 2022, there were 11.55 million health technicians nationwide, including 4.4 million physicians, and 5.2 million registered nurses [13]. The total number of outpatient and emergency visits in the whole year reached 8.4 billion person-times, and the number of discharged patients reached 250 million. China has 27.2 physicians per 10,000 population, lower than the average of 38.3 in central Europe, Eastern Europe, and central Asia [14]. This indicates that Chinese doctors face a heavier workload and more challenging practice environment compared to their Western counterparts. China's healthcare reforms have followed a 'pilot before implementation' approach [15]. However, due to inequalities in healthcare resources across the country [16], reforms that have been effective in developed regions like Beijing and Shanghai may not be as effective in other areas. Rigorous methods are necessary to evaluate the impact and consequences of healthcare reforms. This is essential to determine which policies and interventions are beneficial, ineffective, or even harmful. However, research abroad on the practice environment has largely focused on its influencing factors, such as a study on Norwegian midwives' perceptions of the practice environment [17], and a comparative study on nurses' views of their professional practice environment across seven countries [18]. A similar trend is observed in China, exemplified by a study on Chinese mainland nurses' perspectives on the professional practice environment [19] and a study in Anhui, China, which found that career development, workload, social respect, and monthly income were significant factors affecting the practice environment [5]. These studies have not analyzed the changing in the practice environment and its connection with reforms, overlooking its crucial role as an indicator of the outcomes of healthcare reforms. What’s more, the existing survey mentioned above relies on cross-sectional surveys and lack longitudinal analysis to examine generational differences and changes in long-term practice environments. Generational differences are highly pronounced in Chinese society, with different generations exhibiting varying attitudes and abilities towards their professions [20]. Consequently, a longitudinal study examining medical professionals' perceptions of their practice environment can yield novel insights into past and future reforms.

This study aimed to examine the dynamic trends in Chinese medical practitioners’ perception of their practice work environment from 2008 to 2023 using Age period cohort (APC) analysis. The data were from four repeated national surveys conducted by our team. Compared to other longitudinal research methods that require panel data, the APC model is applicable for repeated cross-sectional data [21]. The APC model is well-suited for analyzing dynamic trends in healthcare professionals' perceptions of their practice environment. This advanced approach can decompose temporal variations into three key sources: age effects reflecting developmental changes over professionals' careers, period effects capturing the influence of external events like healthcare reforms, and cohort effects representing formative experiences shared by professionals born in the same era [22]. By revealing these nuanced age, period, and cohort effects, the study can provide valuable insights that can directly inform tailored policies and initiatives to address the evolving needs of practitioners at various career stages and from different generational backgrounds within the medical workforce impacted by reforms in China. Monitoring such feedback is essential for optimizing and sustaining improvements in the practice environment over time.

## Method

### Study design and participants

In 2008, China had 19,712 hospitals, while at the end of 2022, China had 36,976 hospitals, with the number of medical personnel in those hospitals reaching 8.748 million [13]. The research team conducted four national cross-sectional surveys of Chinese healthcare professionals in 2008, 2013, 2018, and 2023. Multi-stage stratified cluster sampling was applied to select 80 hospitals in 10 provinces in 2008 (*n* = 3,664), 45 hospitals in 9 provinces in 2013 (*n* = 5,852), 45 hospitals in 9 provinces in 2018 (*n* = 11,771), and 40 hospitals in 8 provinces in 2023 (*n* = 8,386). In 2023, the sampling ratio is approximately one in a thousand. China's 31 provincial-level administrative divisions are segmented into eastern, central, and western regions, with varying levels of economic development, the highest in the eastern region and the lowest in the western region. Taking into account the regional disparities in healthcare and socioeconomic development, 2–4 provinces were selected from the eastern, central, and western regions respectively. Additionally, different sample sizes were designed based on the varying number of healthcare professionals in each province. In each province, hospitals were selected proportionally from five different types (provincial hospitals, municipal hospitals, county hospitals, traditional Chinese medicine hospitals, and private hospitals), covering medical institutions of various levels and natures. Within each hospital, different types of healthcare professionals (physicians, nurses, lab technicians, pharmacists and administrators) from different departments (internal medicine, surgery, obstetrics and gynecology, pediatrics, and other departments) were surveyed proportionally based on the proportion of each type of healthcare professionals in the hospital, to ensure balanced representation across occupations and departments. This helps ensure the representative distribution of the research sample in terms of province, hospital type, occupation, and department, to reflect the overall situation of Chinese healthcare professionals. The specific details of the sampling can be found in the supplementary materials and published book [23].

### Data collection

The questionnaires collected information on demographic characteristics, salary and promotion fairness, physical and mental health, job satisfaction, doctor–patient relationship, and perception of the practice environment. These survey questions were kept consistent over the four surveys, conforming to a repeated cross-sectional survey design. Complete questionnaire is in additional files. To ensure the quality of the research, each target province had a designated sub-project leader responsible for the distribution and collection of questionnaires within their respective region. A standardized questionnaire survey manual was developed and followed to facilitate independent distribution and collection of the questionnaires.

### Statistical analysis

Removed 446 outliers or missing values. Perception of the medical practice environment was measured using a 5-point Likert scale (1 = poor, 2 = relatively poor, 3 = fair, 4 = relatively good,5 = good). Age was coded as 18 (< 25) years, 25 (25–34) years, 35 (35–44) years, 45 (45–54) years, and 55 (≥ 55) years, based on the age groups in the questionnaire survey. Age and survey year data were used to generate the variables for age-period-cohort analysis. Age period cohort analyses were performed using R language 4.3.1. The APCtools R package created by Alexander Bauer et al. was utilized, which includes cutting-edge descriptive visualizations as well as visualization and summary functions based on estimating a generalized additive regression model [24]. Unlike other software, APCtools uses a flexible and robust semiparametric regression approach. The package integrates visualization techniques and routines to improve interpretability of temporal structures and simplify APC analysis workflow. More information can be found in the original publication.

To control for the impact of relevant factors, we also included covariates such as sex, department, job satisfaction, position category, salary and promotion fairness, physical and mental health, and DPR.

## Result

### Descriptive analysis

**Fig. 1:**
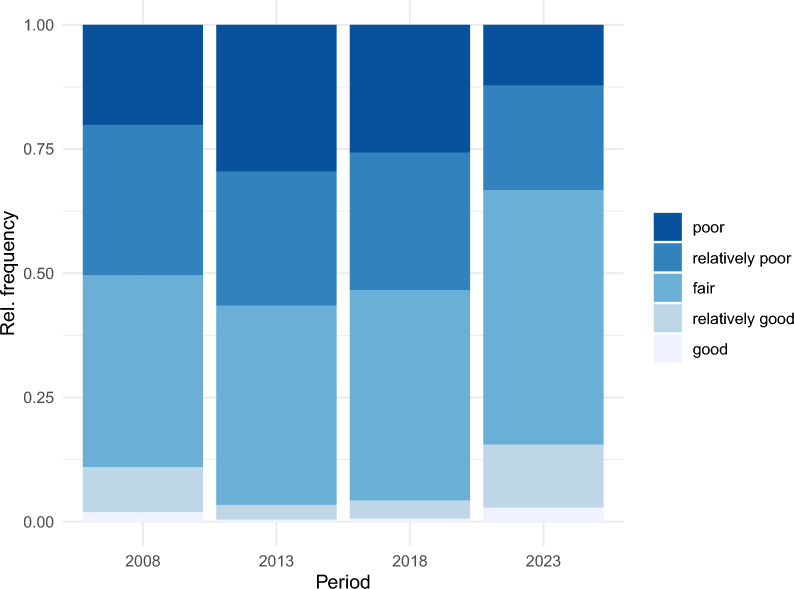
The frequency distribution of healthcare professionals' perceptions of the medical practice environment from 2008 to 2023

Between 2008 and 2023, the frequency distribution of healthcare professionals' perceptions of the medical practice environment shifted, first worsening and then improving (see Fig. 1).

**Fig. 2:**
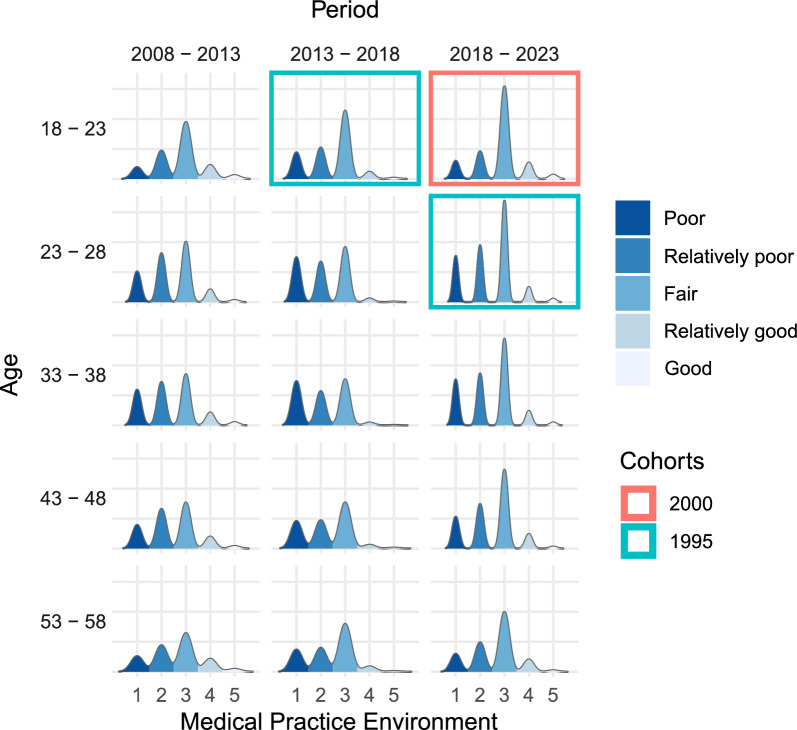
Ridgeline matrix depicts the development of medical practice environment as perceived by healthcare professionals for different age, period, and cohort groups. Two cohort groups are exemplarily highlighted. Higher densities encode higher frequency

The ridgeline matrix displays the changes in medical practitioners' perceptions of their practice environment across different combinations of age, period, and cohort (see Fig. 2).

### Model-based analyses

#### Pure APC model

**Fig. 3:**
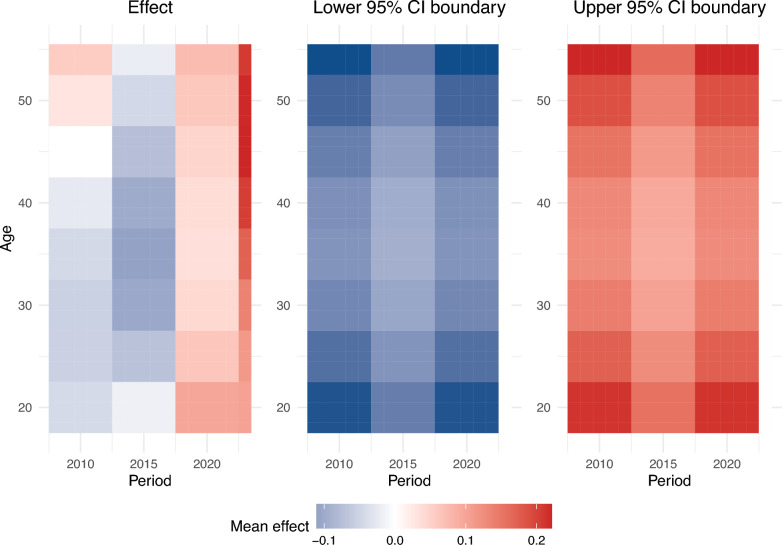
Heatmaps of the estimated tensor product surface (left panel) and the respective lower (center) and upper (right) 95% CI boundary

From the heatmaps, the period between 2013 and 2018 and the age between 28 and 38 years old had the most negative effect on the medical practice environment. The period of 2023 and the age between 43 and 53 years old had the highest positive effect (see Fig. 3).

**Fig. 4:**
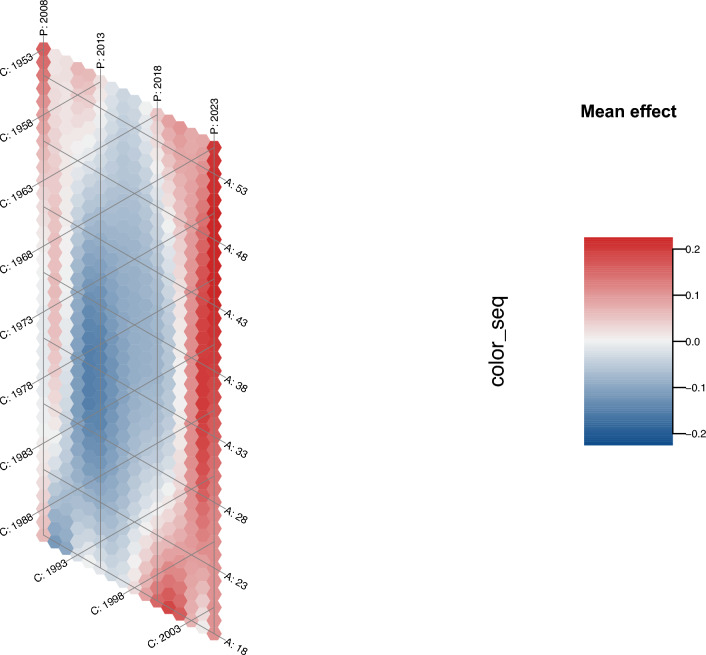
Hexamap of APC mean effect

According to the hexamap of APC mean effects, the period between 2012 and 2013, the age between 28 and 38 years, and the birth cohorts between 1978 and 1983 had the most negative effects (see Fig. 4).

**Fig. 5:**
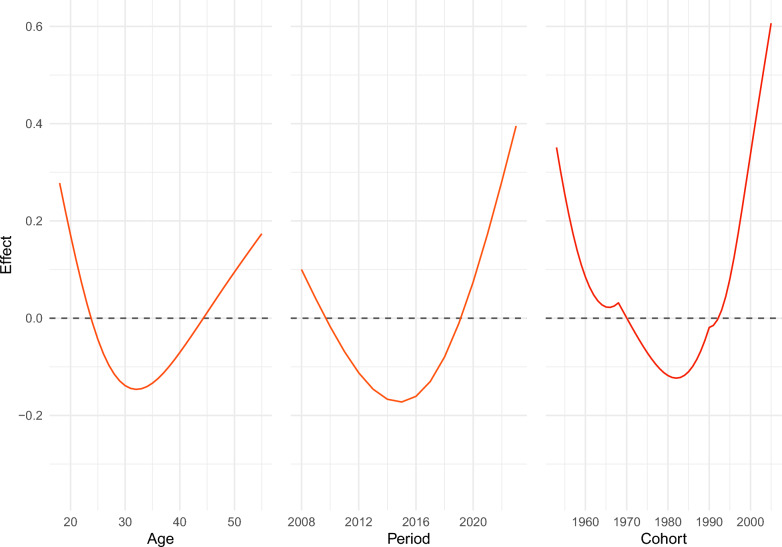
Marginal APC effect

For the marginal age effect, 18 years old was the peak of positive effect, which then decreased, reaching the lowest point of negative effect at 32 years old, and then increased afterwards (see Fig. 5).

For the marginal period effect, the effect decreased from 2008, reaching the lowest point of negative effect in 2015, and then increased, arriving at the highest point of positive effect in 2023 (see Fig. 5).

For the marginal cohort effect, the effect decreased from 1953, reached the lowest point of negative effect in 1982, and then rose, arriving at the peak of positive effect in 2005 (see Fig. 5).

**Fig. 6:**
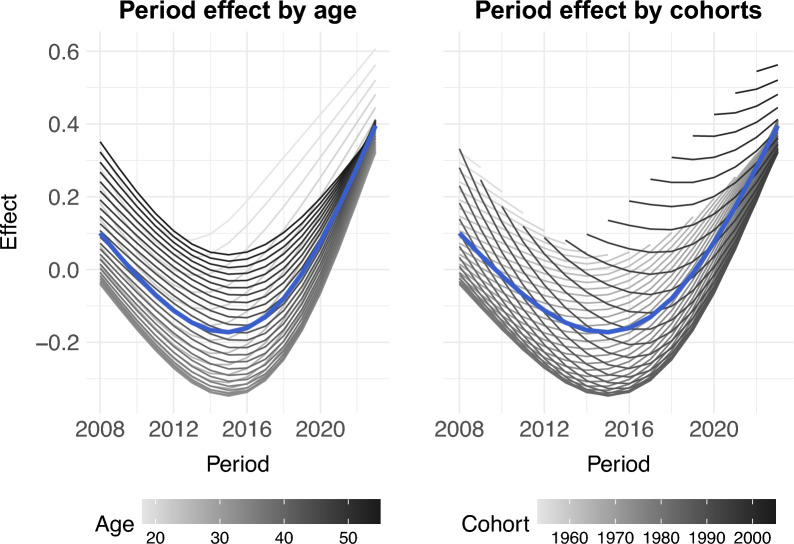
Partial APC plot for the period effect dependent on age group (left panel) and period (right). The mean marginal effect is marked as bold blue line

Figure 6 displays the interactions between period and age, and between period and cohort. The partial period effects separately exhibit the period differences within each age group and cohort. In the left plot, each line represents the period effect at a given age, where the white line (under 20 years old) and black line (over 40 years old) show markedly larger period effects than the grey lines (20–40 years old). In the right plot, each line represents the period effect for a given cohort. For each period, the black line (after 2000) had the most positive influence on the period effect.

Covariate model.

**Fig. 7:**
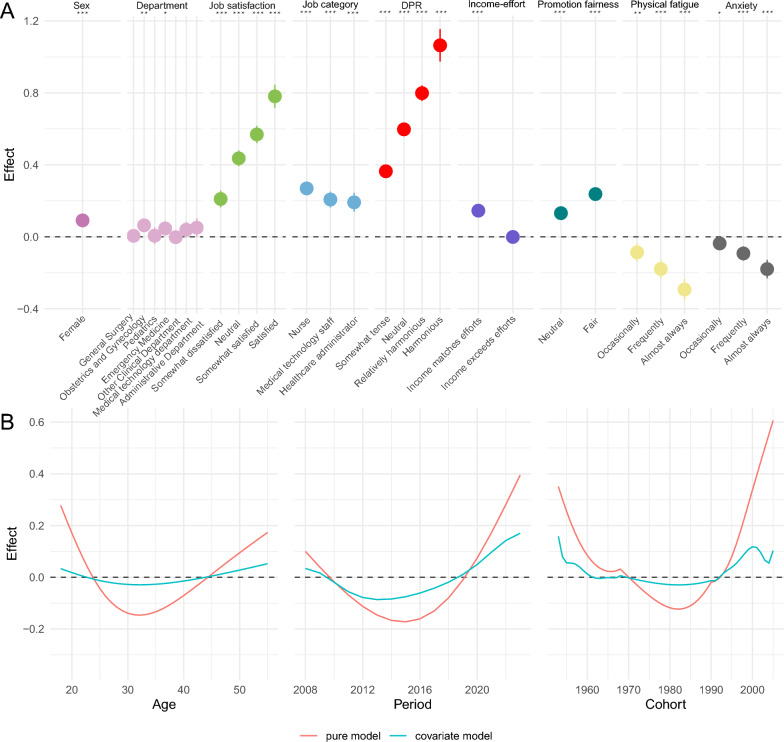
**A** Effects of control variables on the practice environment in the generalized additive regression model. Gender with male as reference group, department with general internal medicine as reference, job satisfaction with unsatisfied as reference, job category with doctor as reference, doctor-patient relationship with tense as reference, income-effort with income lags efforts as the reference, promotion fairness with unfairness as the reference, physical fatigue and anxiety with almost never as the reference (*p < 0.05, **p < 0.01, ***p < 0.0001). **B** The marginal effects on perceived practice environment in the covariate model compared to the effects in the pure APC model. DPR, doctor-patient relationship

In regression results, female has positive effect on perception of practice environment (*p* < 0.0001). In departments, ob-gyn (*p* < 0.01), emergency (*p* < 0.05), have positive effect on perception of practice environment. As job satisfaction and DPR harmony increased, the positive effect on perception of practice environment also increased (*p* < 0.0001). Compared to doctors, the perception of practice environment of nurses, medical technology staff, and healthcare administrators was all better (*p* < 0.0001). Compared to when income lags efforts, when income matches efforts, it has a positive impact on the perception of practice environment (*p* < 0.0001). However, when income exceeds efforts, the positive impact is lost. Healthcare professionals who perceive promotion fairness have a better perception of the practice environment (*p* < 0.0001). The more severe physical fatigue and anxiety are, the more negative impact they have on the perceived practice environment (Fig. 7A).

After controlling for gender, department, job satisfaction and other variables, the curve became flatter, and the strength of APC effects in the model weakened (Fig. 7B). In addition, controlling covariates changed values of age, period, and cohort effects (Table 1).

**Table 1:** The summary table of pure APC model and covariate model

Model	Effect	Value with MaxEffect	Value with MinEffect	Max effect	Min effect	difference
Pure model	Age	18	32	0.28	−0.15	0.42
Pure model	Period	2023	2015	0.40	−0.17	0.57
Pure model	Cohort	2005	1982	0.61	−0.12	0.73
Covariate model	Age	55	32	0.05	−0.03	0.08
Covariate model	Period	2023	2013	0.17	−0.09	0.26
Covariate model	Cohort	1953	1982	0.16	−0.03	0.19

## Discussion

Our study found period effects in Chinese medical practitioners' perceptions of their practice environment from 2008 to 2023 that closely correspond to major healthcare reform initiatives. According to recent researches, a higher job satisfaction often indicates a better practice environment [25, 26]. The decline in perceptions during 2013–2018 aligns with reforms promoting tiered healthcare delivery and diverting patients to community facilities. This indicates that medical personnel in top-ranked hospitals are experiencing a significant degree of income-orientated uncertainty and skepticism in response to disruptive systemic changes that are impacting clinical operations and patient flows [27]. However, in meantime, medical workers in county hospital are experiencing reduced job satisfaction due to surging workloads. Furthermore, they were also skeptical about the effectiveness of healthcare reforms [28]. Our study has yielded similar results to the one previously research in Turkey. Despite patients being satisfied with the reforms, healthcare professionals have consistently complained about their workload being too heavy and their pay being insufficient [6].

The nadir in perceptions in 2015 coincides with early stage reforms like the zero markup drug policy, which sought to curb over-prescription by banning hospitals from profiting on drug sales. While there is a need to rectify distorted incentive mechanisms, it is important to recognize that such corrections could potentially disrupt the revenue streams of hospitals. This, in turn, may have a significant impact on the income levels of healthcare professionals [29], resulting in a temporary deterioration of the practice environment for staff members. Simultaneously, this policy, obviously reduces doctors' level of practice autonomy, particularly among township doctors. The declining income and limited clinical autonomy adversely affect their practice environment, and compels them to leave township hospitals and pursue opportunities for better career advancement in larger healthcare facilities [30].This is also demonstrated by the aforementioned study in Texas. Retention of nurses is influenced by the working environment, and a deteriorating working environment can cause nurses to leave. This has been suggested in a Chinese review published in 2015 [31], and since then a qualitative study abroad has reported similar results, with nurses leaving the clinic because of poor practice environments, high workloads, low pay, and limited clinical autonomy [32]. Ensuring geographic equity in healthcare human resources is important for the sustainable development of a region [33]. The subsequent improvement in perceptions after 2018 relates to a shift in reforms towards measures directly enhancing clinic environments, practice autonomy, career development pipelines, and doctor–patient relationships—addressing more immediate staff concerns.

The peak positive effect seen in 2023 correlates with major reforms in the 2020s to comprehensively transform public hospital management, operations, compensation schemes and governance—as well as new initiatives to resolve healthcare tensions through strengthened legal protections and sanctions against violence towards medical staff. In 2020, the "Basic Medical and Health Promotion Law" was introduced, emphasizing a "zero tolerance" approach towards violence against medical personnel [34]. This indicates that these transformative reforms may be achieving their aims of fundamentally improving the on-the-ground practice environment.

The COVID-19 pandemic beginning in 2020 also exerted period effects on Chinese medical practitioners’ practice environment. Foreign studies have indicated that during the peak of the COVID-19 pandemic, the prevalence of physician burnout reached 57.7% [35]. Furthermore, healthcare professionals who had direct contact with COVID-19 positive patients experienced a significant decrease in job satisfaction [36]. However, in the present study, there has been an observed improvement in healthcare professionals' satisfaction with the medical practice environment from 2020 to 2023. This disparity in findings could potentially be attributed to divergent pandemic response policies between domestic and foreign contexts, with stricter and more organized measures being implemented domestically. Another possible reason is that, during the COVID-19 pandemic, there has been a noticeable shift in public opinion towards Chinese doctors and the doctor–patient relationship [37]. This change may be attributed to media coverage, spontaneously or under government guidance, that has highlighted cases portraying doctors in a positive light [38]. The media has referred to doctors supporting Wuhan as 'countermarch people', praising them for approaching the worst-hit city which others were escaping, in an effort to save more lives [39]. This indicates that in the face of unfavorable internal work factors (high work risks and pressure), enhancing external motivational factors (sense of responsibility, recognition) can also increase healthcare professionals' satisfaction with their practice environment. Utilizing media and other promotional methods to foster positive doctor–patient relationships can inspire a sense of responsibility and recognition among healthcare professionals.

The comprehensive public hospital reforms during 2021–2022 aimed to promote standardized, transparent management and optimized allocation of healthcare resources. New policies also encouraged wider adoption of emerging technologies like artificial intelligence to enhance clinical processes [40]. Although the pandemic increased work pressures, these latest reforms signal continued policy attention to improving the on-the-ground practice environment.

We also found age effects in perceptions, with a peak in early career that began declining from that peak, producing a negative effect starting at age 24, reaching a nadir in the early 30 s, before recovering again in later career stages. There are several potential factors may contribute to this effect. First, empathy is regarded as a significant motivating factor for individuals pursuing a career in medicine [41]. Students opt for medical school due to their inherent empathy, and this empathetic inclination generally remains stable throughout their educational journey [42]. Consequently, newly qualified doctors often exhibit a higher level of enthusiasm and engagement in their professional duties. However, their proficiency in professional knowledge, skills, and business competencies remains limited, leading to various challenges in handling job tasks. These difficulties can easily give rise to feelings of frustration, despondency, and negative emotions, potentially even leading to a loss of confidence [43]. They are confronted with increasing responsibilities and pressures, and without appropriate support and protection, they may risk experiencing burnout and disillusionment in the mid-career phase of their professional journey [44]. To address this issue, it is suggested that reasonable remuneration workload and night shift be provided to those doctors, to improve the expectations of the profession. Hospital managers or senior doctors may be able to provide suitable support for work-related challenges, such as offering regular training [45]. It is recommended that opportunities for continuous professional development and training programs be made available to middle-aged doctors, to enhance their expertise, skills, and professional competence. For example, they could be offered the opportunity to study at higher level hospitals, attend academic conferences, and participate in continuing education courses [46]. These measures have the potential to improve their work performance and increase their confidence in their career prospects. Possible career changes, such as hospital management or research positions, could be made available to those middle-aged [47]. It is important to consider the needs of married doctors, especially female doctors, and provide reasonable time off for marriage and childbearing [26]. It is recommended that pregnancy and childbirth not have a significant impact on their career. A study conducted in Jiangsu, China, revealed a positive association between the duration of medical practice and doctors' remuneration and promotional prospects. Doctors who have accumulated significant practice experience tend to enjoy higher social standing and exhibit greater confidence in their future career growth. Consequently, older doctors typically demonstrate a more optimistic professional mindset compared to their middle-aged counterparts. This positive outlook is so pronounced that it even leads to some medical workers expressing reluctance to retire [48]. For example, subset of retired nurses in the United States choose to resume their careers to reap the following benefits: affirmation of personal value, positive mindset, opportunities for altruistic service, and mitigation of social isolation [49].

According to cohort effects, individuals born after 1995 have consistently demonstrated more favorable perspectives, these findings align with certain research conducted abroad. Internationally, the individuals in question are referred to as "Generation Z" and are considered to possess more open-minded and inclusive perspectives, as well as a greater willingness to embrace novelty. These characteristics are expected to have a positive impact on the medical community [50]. In China, doctors belonging to Generation Z exhibit a more favorable perception of healthcare reform compared to older generations, potentially because they entered the labor market in 2013, after the previous healthcare reform had been implemented. Consequently, they were not directly exposed to the challenges associated with the previous reform and were not negatively affected by it. In contrast, earlier cohorts like the 1970s-1980s exposed to those difficulties exhibited lower perceptions, highlighting how early experiences shape generational outlooks [51]. Our study confirms Mannheim's hypothesis [52] that the socioeconomic and historical events experienced by members of a birth cohort can significantly shape their value orientations. Individuals born in the 1970s and 1980s, also known to researchers as the "social reform generation," are perceived as more pragmatic, more likely to pursue economic success, and more conservative in their political views [20]. Consequently, this generation may have a negative perception of government healthcare reform policies that result in reduced income. Millennials, those born after the 1980s, have faced increased economic pressure due to the one-child policy, which is because that they have to support both parents and children [53]. Negative perceptions are more likely to be shown when their incomes are lower due to healthcare reform. Individuals born in the 1960s hold more positive perceptions of policy. This difference may be attributed to the perception that individuals from the 60 s are more idealistic and collectivist, and have greater confidence in the government [20]. Additionally, those born in the 1960s tended to hold senior positions in the medical field at the time of the survey, with higher incomes and greater respect. As a result, they did not perceive the policy-induced decrease in pay as significantly as others. To enhance perception of the professional environment across different generational cohorts, a multifaceted approach is recommended. For the Generation Z cohort, initiatives should focus on nurturing their innovative mindsets and embracing inclusivity through specialized training programs. Establishing feedback mechanisms to amplify their voices is crucial. Addressing the pragmatic concerns of the social reform generation necessitates safeguarding reasonable income levels, offering professional development and retraining avenues. The economic pressures faced by millennials, exacerbated by the one-child policy, should be alleviated through measures such as housing and child education support, enhanced compensation packages, and work-life balance policies.

Controlling for covariates revealed that female practitioners as well as those in obstetrics and emergency medicine had more positive perceptions of the practice environment. Higher job satisfaction and more harmonious doctor–patient relationships are also associated with more favorable perceptions. A similar result was shown by Hou et al. [54]. Compared to physicians, nurses, technicians and administrators also had more positive perceptions. This study is comparable to research conducted in Israel, where female nurses and working in emergency reported higher levels of job satisfaction [55]. Furthermore, as stated in the Norwegian study, even among professionals in the same field, midwives who do not perform managerial duties have a less-favorable evaluation of their work environment than those who do [17]. The alignment between income and efforts also played a crucial role. When income matched efforts, it positively impacted perceptions of the practice environment compared to situations where income lagged behind efforts. However, this positive impact diminished when income exceeded efforts, suggesting that overly high compensation may not necessarily translate to improved perceptions. This finding is noteworthy, as it aligns with a previous study on the relationship between income and happiness that reached a similar conclusion [56]. Furthermore, perceptions of promotion fairness were linked to more positive views of the practice environment, while more severe physical fatigue and anxiety negatively affected these perceptions. This statement is consistent with the findings of a qualitative study conducted in Iran [57]. To enhance healthcare providers' perceptions of the practice environment, an approach targeting various interrelated factors may be beneficial.

## Conclusion

In conclusion, healthcare professionals' perceptions of their practice environment first declined and then improved in 2008–2023. Those aged 28–38 during 2013–2018 and born between 1978 and 1988 had the most negative perceptions. Females, those in obstetrics and emergency medicine, nurses, technicians, and administrators perceived better environments. Higher job satisfaction and doctor–patient relationship harmony also associated with more positive perceptions. Income matching efforts and perceptions of promotion fairness had positive impacts, while increasing severity of physical fatigue and psychological anxiety negatively influenced perceptions of the practice environment. The study reveals intertwined effects on perceptions across age, periods, and generations, thereby enhancing theoretical understanding of how healthcare policy and system changes intersect with individual development and cohort identities to shape perspectives.

## Supplementary Information

Supplementary material 1.

Supplementary material 2.

Supplementary material 3.

Supplementary material 4.

## Data Availability

The datasets generated and analyzed during the current study are not publicly available due to privacy and confidentiality concerns, but are available from the corresponding author on reasonable request.

## Abbreviations

APC: Age-period-cohort
DPR: Doctor-patient relationship
