# Supplementary material for: Trends in the practice environment of Chinese healthcare professionals from 2008 to 2023: an age period cohort analysis
Source: Hum Resour Health. 2024 Nov 13;22:76. doi: 10.1186/s12960-024-00954-5 (PMC11562610; doi:10.1186/s12960-024-00954-5)
Supplement: Supplementary file 1 — Supplementary material 1. [file 12960_2024_954_MOESM1_ESM.docx]

Survey of Employment Status of Medical Workers in 10 Provinces and Cities

Dear colleagues:

Entrusted by the Publicity Department of the Chinese Association of Science and Technology, the Chinese Academy of Medical Sciences/Peking Union Medical College is conducting a survey on the "Employment Status of Medical Workers". This survey will be carried out simultaneously in more than 70 hospitals in 10 provinces across the country, to understand the practice environment and conditions, as well as the attitudes of medical workers in China. This survey is anonymous and your participation is voluntary. Incomplete questionnaires will not be included in the analysis. It takes about 20 minutes to complete this questionnaire. Please answer the questions as required. Return or mail the completed questionnaire to the distributor.

I. Your personal basic information

1.1 Gender: 1 Male 2 Female

1.2 Age: ________

1.3 Highest education: 1 Secondary vocational 2 Associate degree 3 Bachelor’s degree 4 Graduate degree 5 Other

1.4 Professional title: 1 Junior 2 Intermediate 3 Associate senior 4 Senior 5 Unrated

1.5 Technical position: 1 Physician 2 Nurse 3 Medical technician 4 Health administrator 5 Other

1.6 Monthly salary (including wages and bonuses): 1 ≤1000 2 1001-2000 3 2001-3000 4 3001-4000 5 4001-5000 6 5001-6000 7 6001-7000 8 ≥7000

1.7 Type of hospital: 1 Public general hospital 2 Public specialized hospital 3 TCM hospital 4 Private hospital 5 Community health center 6 Township health center 7 Other

1.8 Hospital grade: 1 Grade 3A 2 Grade 3B 3 Grade 2A 4 Grade 2B 5 Grade 1

1.9 Department: 1 Internal medicine (Specify: ____) 2 Surgery (Specify: ____) 3 Obstetrics and Gynecology 4 Pediatrics 5 Emergency Medicine 6 Other clinical departments 7 Auxiliary departments 8 Administration

1.10 Years in medical practice: _______

1.11 Province: _______

II. Your feelings about work pressure and occupational risks

2.1 Average daily working hours: 1 ≤6 2 7 3 8 4 9 5 10 6 ≥11

2.2 My current busy workload makes me feel: 1 Interested 2 Relaxed 3 Tired 4 Exhausted

2.3 I often have to do things against my conscience at work: 1 Agree 2 Disagree

2.4 In addition to routine medical activities, are you involved in: (Multiple choice)

1 Teaching 2 Research 3 Administration 4 Part-time job 5 None of the above

2.5 Please evaluate your overall work risks (Single choice):

1 Too high 2 High 3 General 4 Low 5 Almost none

See next page

2.6 In the past year, how many times have you been pierced by medical sharps (such as needles, scalpel blades) during medical activities? (Single choice)

1 Never 2 1-3 times 3 4-6 times 4 7-8 times 5 >8 times

2.7 In your daily medical work, what are your major concerns? (Choose up to 3 items)

1 Medical errors 2 Patient complaints 3 No time for family

4 Night shifts 5 Bleak prospects 6 Health hazards from hospital environment

7 Strained colleague relationships 8 Work overload 9 Lack of knowledge and skills

2.8 In the past month, to what extent did you experience the following?

Options: Almost never Rarely Often Almost always

1) Physical fatigue, discomfort 1 2 3 4

2) Forcing myself to do unwanted things 1 2 3 4

3) Gloom, loss of interest, pessimism 1 2 3 4

4) Anxiety, irritability, nervousness 1 2 3 4

2.9 Do your colleagues avoid high-risk surgeries for fear of medical disputes?

1 Always 2 Often 3 Sometimes 4 Rarely 5 Never

2.10 Is nursing care given due attention in your hospital? 1 Yes 2 No

III. Your views on remuneration fairness

3.1 Estimate the relationship between your remuneration (including wages and bonuses) and your work contribution: (Single choice)

1 Income > Contribution 2 Income = Contribution 3 Income < Contribution 4 Income << Contribution

3.2 Compared to your current contribution, your ideal monthly salary (including wages and bonuses) is: (Single choice)

1 ≤2000 2 2001-3000 3 3001-4000 4 4001-5000 5 5001-6000

6 6001-7000 7 7001-8000 8 8001-9000 9 ≥9000

3.3 Do you agree that relying solely on wages (including bonuses) makes it difficult to maintain livelihood: (Single choice)

1 Strongly agree 2 Agree 3 No opinion 4 Disagree 5 Strongly disagree

3.4 Imagine a medical worker feeling underpaid relative to their work contribution, and is able to receive red packets or kickbacks. To what extent can they still practice with "integrity and devotion"? (Single choice)

1 Fully able 2 Largely able 3 Sometimes able 4 Barely able 5 Completely unable

3.5 When pursuing economic efficiency becomes the top priority of a hospital, to what extent can its medical staff still "always think of the patients, and do everything possible to relieve their suffering"? (Single choice)

1 Fully able 2 Largely able 3 Sometimes able 4 Barely able 5 Completely unable

3.6 Does your current income reflect factors like your technical services and risks?

1 Fully reflects 2 Mainly reflects 3 Partially reflects 4 Hardly reflects

3.7 To reflect the value of doctors' services, outpatient visit fees should be substantially increased: 1 Agree 2 Disagree 3 Indifferent

IV. Your assessment of career development status and prospects

4.1 What motivated you to initially choose this profession? (Single choice)

1 Voluntarily 2 Parents or friends wished 3 Unconsciously 4 Other

4.2 What is your top priority for career development in the next few years? (Multiple choice)

1 No specific plan 2 Obtain professional certification 3 Further studies or degree

4 Participate in research projects 5 Improve clinical skills 6 Improve humanities literacy and moral character

See next page

4.3 If you had the chance to choose again, would you still choose your current job? 1 Yes 2 No

4.4 Do you hope your children will become doctors in the future? 1 Yes 2 No

4.5 If considering leaving your job in the future, which three factors would you consider? (Choose up to 3 items)

1 Income and benefits 2 Hospital prospects 3 Social recognition

4 Personal development space 5 Workload and tension 6 Occupational risks

7 Regularity of work hours 8 Interpersonal relationships 9 Other (please specify):

4.6 Your opinion on the fairness of professional title promotion is: (Single choice)

1 Fair 2 Quite fair 3 No opinion 4 Quite unfair 5 Unfair

4.7 You believe the most important aspect considered in professional title evaluation is: (Single choice)

1 Foreign language proficiency 2 Research capability 3 Theoretical level 4 Clinical skills

5 Years of service 6 Moral character 7 Other (please specify):

4.8 Can your current position fully utilize your capabilities? 1 Yes 2 No

4.9 To what extent does your superior (or department director) pay attention to talent pipeline building?

1 Very attentive 2 Attentive 3 General 4 Inattentive 5 Very inattentive

4.10 Does the hospital provide convenience for your continuing medical education? 1 Yes 2 No

4.11 How satisfied are you with your current position overall? (Single choice)

1 Very satisfied 2 Satisfied 3 General 4 Dissatisfied 5 Very dissatisfied

4.12 Your view of your current profession is: 1 Sacred profession 2 Honorable profession 3 Livelihood 4 Lowly profession

V. Your understanding and views on doctor-patient relationships

5.1 How is the overall doctor-patient relationship in your hospital currently? (Single choice)

1 Very tense 2 Tense 3 General 4 Harmonious 5 Very harmonious

5.2 Currently, how much do patients trust you? (Single choice)

1 Very trusting 2 Trusting 3 General 4 Distrusting 5 Very distrusting

5.3 How much do patients respect you? (Single choice)

1 Respect 2 Quite respect 3 Disrespect 4 Very disrespect

5.4 In actual medical practice, to what extent are patients involved in clinical decisions? (Single choice)

1 Most cases 2 Some cases 3 Few cases 4 Never

5.5 The close contact with patients in medical work gives me a sense of accomplishment:

1 Always 2 Often 3 Sometimes 4 Rarely 5 Never

5.6 Has your rights and interests been infringed by patients or their families in the past year? (Single choice)

Options: None 1-2 times 3-4 times ≥5 times

1) Injured by patients 1 2 3 4

2) Insulted by patients 1 2 3 4

3) Threatened by patients 1 2 3 4

5.7 In your hospital for example, the fundamental causes of doctor-patient conflicts arising from the medical side are: (Choose up to 2 items)

1 Poor doctor-patient communication 2 Over-prescription or over-examination

3 Negligence, misdiagnosis or mistreatment 4 Uncertainty or limitations of medicine

5 Poor service attitude 6 Other (please specify):

5.8 You believe the initiative in properly resolving doctor-patient disputes lies with: 1 Doctors 2 Patients 3 Both sides

5.9 The regulation of "reversed burden of proof" makes me very cautious when working to avoid doctor-patient disputes:

1 Always 2 Often 3 Sometimes 4 Rarely 5 Never

5.10 The impact of giving red packets on medical staff's service attitude is: 1 Major 2 Minor

See next page

5.11 Imagine a hospital received a patient in urgent need of surgery. After being fully informed of the risks and benefits, the patient's family refuses to sign the consent form. What should the attending doctor do? (Single choice)

1 Operate on the patient immediately

2 Give up operating on the patient, take conservative treatment

3 Submit to hospital management or health authorities for deliberation

4 Other (please specify): ________________

VI. Your cognition and response to the practice environment

6.1 Your overall evaluation of the current practice environment for medical workers in China is: (Single choice)

1 Good 2 Quite good 3 General 4 Quite poor 5 Poor

6.2 The atmosphere of "teaching, helping and guiding" talents in your hospital is: (Single choice)

1 Very good 2 Good 3 General 4 Poor 5 Very bad

6.3 Your family and friends' attitude towards your current profession is: (Single choice)

1 Very satisfied 2 Satisfied 3 General 4 Dissatisfied 5 Very dissatisfied

6.4 Your evaluation of the medical equipment needed for your current work:

1 Very satisfied 2 Satisfied 3 General 4 Dissatisfied 5 Very dissatisfied

6.5 Does media publicity vilify the image of medical workers? (Single choice)

1 Most cases 2 Some cases 3 Few cases 4 Very few cases

6.6 The attitude of most media in reporting medical dispute cases is: (Single choice)

1 Biased towards patients 2 Biased towards doctors 3 Objective and impartial

6.7 Please evaluate whether your hospital has a culture that encourages innovation: (Single choice)

1 Very good 2 Good 3 General 4 Poor 5 Very bad

6.8 Has your unit carried out "Hospital Administration Year" activities? 1 Yes 2 No 3 Unsure

6.9 Are you willing to transfer recovering patients from your hospital to other hospitals: 1 Yes 2 No 3 Unsure

6.10 The impact of applying "top-level, refined and sophisticated" diagnostic equipment clinically on doctor-patient communication is: 1 Positive 2 Negative

6.11 Your attitude towards reforming your hospital's internal management mechanism is:

1 Very urgent 2 Needs reform 3 Can wait longer 4 Unnecessary

6.12 What kind of ownership reform do you think is appropriate for public hospitals: (Single choice)

1 State-holding shareholding 2 Convert to non-public hospital 3 State and private joint-operation

4 Maintain current ownership structure unchanged 5 Other (please specify):

Thank you for your participation!

VI. Your cognition and response to the practice environment (TCM hospitals)

6.1 Your overall evaluation of your own practice environment is: (Single choice)

1 Good 2 Quite good 3 General 4 Quite poor 5 Poor

6.2 The atmosphere of "teaching, helping and guiding" talents in your hospital is: (Single choice)

1 Very good 2 Good 3 General 4 Poor 5 Very bad

6.3 Your family and friends' attitude towards your current profession is: (Single choice)

1 Very satisfied 2 Satisfied 3 General 4 Dissatisfied 5 Very dissatisfied

6.4 Does media publicity vilify the image of medical workers? (Single choice)

1 Most cases 2 Some cases 3 Few cases 4 Very few cases

6.5 The attitude of most media in reporting medical dispute cases is: (Single choice)

1 Biased towards patients 2 Biased towards doctors 3 Objective and impartial

6.6 Is your hospital lacking in backup talents: 1 Yes 2 No

6.7 Which factor restricts the "utilization of TCM's strengths" the most? (Single choice)

1 Patients don't recognize it 2 Unclear policy orientation 3 Poor efficacy 4 Poor economic benefits 5 Hospital management concepts

6.8 What is the current state of integration of Traditional Chinese Medicine and Western Medicine: 1 Theoretical integration 2 Applied integration 3 Neither

6.9 Do you agree with introducing modern diagnostic techniques into TCM hospitals: 1 Agree 2 Disagree

6.10 Is the advantage of traditional TCM disappearing amid the development of modern medicine: 1 Yes 2 No

6.11 Under the current medical system, the direction TCM hospitals should take is: (Single choice)

1 Develop pure TCM therapies 2 Mainly TCM, combined with Western medicine 3 Mainly Western medicine, TCM as complement

VI. Your cognition and response to the practice environment (Private hospitals)

6.1 Your overall evaluation of your own practice environment is: (Single choice)

1 Good 2 Quite good 3 General 4 Quite poor 5 Poor

6.2 The atmosphere of "teaching, helping and guiding" talents in your hospital is: (Single choice)

1 Very good 2 Good 3 General 4 Poor 5 Very bad

6.3 Your family and friends' attitude towards your current profession is: (Single choice)

1 Very satisfied 2 Satisfied 3 General 4 Dissatisfied 5 Very dissatisfied

6.4 Does media publicity vilify the image of medical workers? (Single choice)

1 Most cases 2 Some cases 3 Few cases 4 Very few cases

6.5 The attitude of most media in reporting medical dispute cases is: (Single choice)

1 Biased towards patients 2 Biased towards doctors 3 Objective and impartial

6.6 Is your hospital lacking in backup talents: 1 Yes 2 No

6.7 You believe the biggest difficulty faced by private hospitals is: (Single choice)

1 Difficult approval and business restrictions 2 Fundraising difficulties 3 Unhealthy competition from peers

4 Lack and high turnover of talents 5 Heavy taxes 6 Credibility crisis 7 Other (please specify)

6.8 Compared to public hospitals, what do you value most about private hospitals: (Multiple choice)

1 Flexible talent utilization 2 Higher income 3 Comfortable work environment

4 Competitive pressure 5 Able to utilize professional capabilities 6 Standardized hospital management 7 None of the above

6.9 Your opinion on the market competition environment for private hospitals is: (Single choice)

1 Fair 2 Quite fair 3 No opinion 4 Quite unfair 5 Unfair

VI. Your cognition and response to the practice environment (Primary hospitals)

6.1 Your overall evaluation of your own practice environment is: (Single choice)

1 Good 2 Quite good 3 General 4 Quite poor 5 Poor

6.2 The atmosphere of "teaching, helping and guiding" talents in your hospital is: (Single choice)

1 Very good 2 Good 3 General 4 Poor 5 Very bad

6.3 Your family and friends' attitude towards your current profession is: (Single choice)

1 Very satisfied 2 Satisfied 3 General 4 Dissatisfied 5 Very dissatisfied

6.4 Is the positioning of primary hospitals clear under socialist market economy conditions: 1 Yes 2 No

6.5 Can current primary hospitals resolve patients' medical needs: 1 Yes 2 No

6.6 Why are college graduates unwilling to work in primary hospitals?

1 Low income 2 Hospitals unwilling to hire 3 Poor work environment 4 Little development space 5 Other (please specify)

6.7 Are you satisfied with the current implementation of two-way referral system:

1 Very satisfied 2 Satisfied 3 General 4 Dissatisfied 5 Very dissatisfied

6.8 Do large hospitals currently transfer patients with common diseases, prevalent diseases and recovering patients to community hospitals and township hospitals?

1 Most cases 2 Some cases 3 Few cases 4 Very few cases

6.9 Do you agree with Grade 3 hospitals operating community hospitals when necessary: 1 Agree 2 Disagree 3 Unsure

6.10 Under the two-way referral system, can the "primary care physician in charge" system be implemented in community hospitals: 1 Yes 2 No

Thank you for your participation!
