# Supplementary material for: Trends in the practice environment of Chinese healthcare professionals from 2008 to 2023: an age period cohort analysis
Source: Hum Resour Health. 2024 Nov 13;22:76. doi: 10.1186/s12960-024-00954-5 (PMC11562610; doi:10.1186/s12960-024-00954-5)
Supplement: Supplementary file 2 — Supplementary material 2. [file 12960_2024_954_MOESM2_ESM.docx]

**Survey of Employment Status of Medical Personnel in 9 Provinces and Cities**

**(Public General Hospital Section)**

Entrusted by the Publicity Department of the Chinese Association for Science and Technology, the Chinese Academy of Medical Sciences/Peking Union Medical College has taken the lead in conducting this questionnaire survey. By understanding the employment status of medical personnel since the new medical reform, information will be provided for national decision-making. The survey is anonymous and voluntary. It takes about **20** minutes to fill out the questionnaire. Please tick "**√**" on the options. The vast majority of questions are single-choice unless otherwise noted. Thank you for your cooperation!

**I. Personal Information**

1.1 Gender: **1 Male 2 Female**

1.2 Age: **1 <25 2 25-34 3 35-44 4 45-54 5 >54**

1.3 Highest Education: **1 Secondary vocational school or below 2 Associate degree 3 Bachelor's degree 4 Graduate degree**

1.4 Professional Title: **1 Junior 2 Intermediate 3 Associate Senior 4 Senior 5 Unrated**

1.5 Technical Position: **1 Physician 2 Nurse 3 Medical technician/Pharmacist 4 Administrator**

1.6 Monthly Income: **1 <2000 2 2001-4000 3 4001-6000 4 6001-8000 5 >8000 RMB**

1.7 Hospital Type: **1 Grade 3A General 2 Grade 2A General 3 TCM Hospital 4 Private Hospital**

1.8 Department: **1 Internal Medicine 2 Surgery 3 Obstetrics and Gynecology 4 Pediatrics 5 Emergency Medicine**

**6 Other Clinical Departments 7 Medical Technology Departments 8 Administration Departments**

1.9 Employment Type: **1 On payroll 2 Not on payroll**

1.10 Province: **1 Beijing 2 Jiangsu 3 Guangdong 4 Liaoning 5 Henan 6 Hunan 7 Yunnan 8 Shaanxi 9 Xinjiang**

**II. Work Pressure and Physical and Mental Health**

2.1 How much **work pressure** do you feel: **1 Very little 2 Little 3 General 4 High 5 Very high**

2.2 Your average daily **working hours** (**hours**): **1 <8 2 8 3 9 4 10 5 >10**

2.3 Compared to the current workload, the **staffing** of medical personnel in this department is: **1 Insufficient 2 Appropriate 3 Excessive**

2.4 Are you involved in the following **activities** or work? (Multiple choice)

**1 Scientific research 2 Teaching 3 Management 4 External hospital consultation 5 Multi-site practice 6 None of the above**

2.5 Your main sources of **pressure** currently are: (Within 3 items)

**1 Medical errors 2 Patient complaints 3 Low income and benefits 4 Overtime work, night shifts 5 Bleak prospects 6 Health damage 7 Strained interpersonal relationships 8 Heavy workload 9 Lack of knowledge and skills 10 Other**

2.6 In the past month, how often did you experience symptoms like "**physical fatigue, discomfort**", etc:

**1 Almost never 2 Rarely 3 Often 4 Almost always**

2.7 In the past month, how often did you experience symptoms like "**tension, nervousness, restlessness or irritability**":

**1 Almost never 2 Rarely 3 Often 4 Almost always**

2.8 In the past month, how often did you experience symptoms like "**lack of energy, difficulty making decisions or need to recheck things repeatedly**":

**1 Almost never 2 Rarely 3 Often 4 Almost always**

2.9 In the past month, how often did you experience symptoms like "**gloom, loss of interest, pessimism or easy crying**":

**1 Almost never 2 Rarely 3 Often 4 Almost always**

**III. Job Satisfaction and Turnover Intention**

3.1 How satisfied are you with your current job position **overall**:

**1 Very dissatisfied 2 Dissatisfied 3 General 4 Satisfied 5 Very satisfied**

3.2 How is the relationship between your **remuneration** (including salary and bonuses) and your own work contribution:

**1 Contribution > Income 2 Income = Contribution 3 Contribution < Income**

3.3 You feel your annual **total income** should reach \_\_\_\_\_ ten thousand RMB?

3.4 Do you think medical staff can still practice "**clean medicine**" when they are able to receive "**red packets or kickbacks**"?

**1 Basically cannot 2 Sometimes can 3 Basically can**

3.5 In your opinion, what is the fundamental factor inducing medical staff to accept "**red packets, kickbacks**"?

**1 Poor personal conduct 2 Low income 3 Supporting healthcare with medicine sales 4 Industry unspoken rules 5 Other**

3.6 How fair do you think the promotion of technical **professional titles** is: **1 Unfair 2 No opinion 3 Fair**

3.7 If you had the opportunity to **choose your career again**, would you still choose your current profession? **1 No 2 Yes 3 Unsure**

3.8 Do you hope your **children will study medicine**: **1 No 2 Yes 3 Unsure**

3.9 Your **evaluation** of your current **profession** is: **1 Sacred 2 Valuable 3 Livelihood 4 Low profession**

**IV. Rights Protection and Career Development**

4.1 The protection of your legitimate **rights and interests** in practice is: **1 Poor 2 General 3 Good**

4.2 Do you have **medical liability insurance**: **1 No 2 Yes 3 Unsure**

4.3 The **talent pipeline** building in this department is: **1 Poor 2 General 3 Good**

4.4 The degree to which your **abilities are utilized** in your current position is: **1 Poor 2 General 3 Good**

4.5 Does the hospital provide you with convenience for **continuing education**: **1 Not provided 2 Provided**

4.6 Is the phenomenon of **"needed people unable to enter, redundant people unable to leave"** serious in this unit: **1 Not serious 2 Serious**

4.7 What is your top priority for **career development** in the next few years? (Within 3 items)

**1 No specific plan 2 Obtain professional certification 3 Further studies or degree 4 Participate in scientific research**

**5 Improve operational skills 6 Improve humanities literacy 7 Other**

4.8 Can **public hospital reform** provide you with greater **career development opportunities**: **1 No 2 Yes 3 Unsure**

**V. Doctor-Patient Relationship and Medical Ethics**

5.1 You feel the current **doctor-patient relationship** is: **1 Very tense 2 Tense 3 General 4 Harmonious 5 Very harmonious**

5.2 Last year, the **number of times** you suffered "**verbal abuse**" from patients was: **1 0 2 1-2 3 3-4 4 >4**

5.3 Last year, the **number of times** you had "**physical conflicts**" with patients was: **1 0 2 1-2 3 >3**

5.4 The degree of **trust** patients have in you is: **1 Distrust 2 General 3 Trust**

5.5 You believe the prominent manifestations of **doctor-patient disputes** caused by the **medical side** are: (Within 3 items)

**1 Poor doctor-patient communication 2 Over-prescription or over-examination 3 Missed diagnosis, misdiagnosis 4 Limitations of medicine**

**5 Poor service attitude 6 Heavy work pressure 7 Other**

5.6 You believe the **best** way to properly resolve **doctor-patient disputes** is:

**1 Doctor-patient negotiation 2 Litigation 3 Third party mediation 4 Administrative mediation 5 Other**

5.7 You believe the fundamental causes of "**overtreatment**" are (Within 3 items):

**1 Complex patient conditions 2 Unreasonable patient demands 3 Temptation of kickbacks and commissions 4 Poor medical ethics**

**5 Doctor's diagnostic and treatment preferences 6 Doctor's diagnostic and treatment skills 7 Other**

5.8 Facing doctor-patient **conflicts of interest**, whose interest is placed first: **1 Patient's interest 2 Hospital's interest 3 Personal interest**

5.9 Imagine a critically ill patient urgently needing surgery, but the family is fully informed yet still **refuses** to **sign** the consent form. You believe the attending doctor should first:

**1 Operate on the patient immediately**

**2 Give up operating on the patient, take conservative treatment**

**3 Submit to hospital or supervising authority for deliberation**

**4 Other**

**VI. Practice Environment and Hospital Reform**

6.1 The overall status of China's current medical **practice environment** is: **1 Very poor 2 Poor 3 General 4 Good 5 Very good**

6.2 Does **media public opinion** vilify the image of medical staff: **1 Few instances 2 Some instances 3 Most instances**

6.3 Is **media coverage** biased towards patients when reporting medical disputes: **1 Occasionally 2 Sometimes 3 Always**

6.4 How do you feel about the implementation of the goals to "**ensure basic healthcare, strengthen grassroots healthcare, and build mechanisms**" in the current new medical reforms:

**1 Not achieved 2 Partially achieved 3 Largely achieved 4 Hard to say**

6.5 Will breaking "**supporting healthcare with medicine sales**" lead to your income and benefits decreasing: **1 No 2 Yes 3 Hard to say**

6.6 The biggest **obstacle** in breaking the pattern of "**supporting healthcare with medicine sales**" comes from (Within 2 items):

**1 Hospitals 2 Price control departments 3 Medical insurance departments 4 Finance departments 5 Pharmaceutical companies 6 Other**

6.7 You feel the main difficulties encountered in currently implementing the **tiered healthcare** system are (Within 3 items):

**1 Inadequate supervision 2 Lack of patient cooperation 3 Poor referral information flow 4 Conflicts of interest**

**5 Hard to grasp referral criteria 6 Lack of primary care personnel 7 Other**

6.8 The prominent issues in your hospital's current **cultural development** are (Within 2 items):

**1 Unclear value orientation 2 Becoming a formality 3 Insufficient funding 4 Leadership does not value it 5 Other**

6.9 You believe the most prominent causes of **medical resource waste** in China are (Within 3 items):

**1 Unnecessary medical services 2 High management costs 3 Low service efficiency 4 Soaring prices**

**5 Medical fraud 6 Neglecting prevention 7 Other**

6.10 To meet the ever-increasing healthcare needs, this hospital should **expand** its scale: **1 Oppose 2 Agree 3 Unsure**

6.11 Do you agree with your hospital participating in building **regional healthcare consortia**: **1 Disagree 2 Partially agree 3 Largely agree

6.12 Do you have any suggestions for improving the working conditions and environment for public hospital staff:

Thank you for your participation!**
