# Supplementary material for: Trends in the practice environment of Chinese healthcare professionals from 2008 to 2023: an age period cohort analysis
Source: Hum Resour Health. 2024 Nov 13;22:76. doi: 10.1186/s12960-024-00954-5 (PMC11562610; doi:10.1186/s12960-024-00954-5)
Supplement: Supplementary file 3 — Supplementary material 3. [file 12960_2024_954_MOESM3_ESM.docx]

National Survey of Medical Personnel Employment Status

Entrusted by the Publicity Department of the Chinese Association for Science and Technology, the Chinese Academy of Medical Sciences/Peking Union Medical College has taken the lead in conducting questionnaire surveys in 45 hospitals across 9 provinces. By understanding the employment status of medical personnel since the new medical reform, information will be provided for national decision-making. The survey is anonymous and voluntary. It takes about 10 minutes to fill out the questionnaire. Please tick "√" on the options. Most questions are single choice or choose three options, unless otherwise noted. Thank you for your cooperation!

I. Personal Information

1.1 Gender: 1 Male 2 Female

1.2 Age: 1 <25 2 25-34 3 35-44 4 45-54 5 >54

1.3 Highest Education: 1 Secondary vocational school or below 2 Associate degree 3 Bachelor's degree 4 Master's degree 5 Doctoral degree

1.4 Professional Title: 1 Junior 2 Intermediate 3 Associate Senior 4 Senior 5 Unrated

1.5 Technical Position: 1 Physician 2 Nurse 3 Medical technician/Pharmacist 4 Administrator

1.6 Monthly Income (RMB): 1 <2000 2 2001-4000 3 4001-6000 4 6001-8000 5 8001-10000 6 10001-12000 7 >12000

1.7 Hospital Type: 1 Provincial general hospital 2 Municipal general hospital 3 County people’s hospital 4 Traditional Chinese medicine hospital 5 Private hospital

1.8 Hospital Grade: 1 Grade 3A 2 Grade 3B 3 Grade 2A 4 Grade 2B 5 Unrated

1.9 Department: 1 Internal medicine 2 Surgery 3 Obstetrics and Gynecology 4 Pediatrics 5 Emergency 6 Anesthesiology 7 Other clinical departments 8 Medical technology/Pharmacy 9 Administration

1.10 Employment Type: 1 On payroll 2 Not on payroll

1.11 Province: 1 Beijing 2 Liaoning 3 Henan 4 Shaanxi 5 Ningxia 6 Sichuan 7 Guangxi 8 Jiangsu 9 Guangdong

II. Work, Life and Physical and Mental Health

2.1 Your average daily working hours: 1 <8 2 8 3 9 4 10 5 >10

2.2 Compared to the current workload, the staffing of medical personnel in this department is: 1 Insufficient 2 Appropriate 3 Excessive

2.3 Are you involved in the following work? (Multiple choice)

1 Scientific research 2 Teaching 3 Management 4 External hospital consultation 5 Multi-site practice 6 None

2.4 How much work pressure do you feel: 1 Very little 2 Little 3 General 4 High 5 Very high

2.5 What do you think are the outstanding issues medical staff encounter when conducting scientific research? (Choose 3)

1 Heavy clinical workload 2 Research divorced from clinical practice 3 Lack of research team 4 Lack of research capability 5 Lack of research interest 6 Lack of research funding 7 Cumbersome reimbursement procedures 8 Other (note: _____)

2.6 What are your main sources of work pressure currently? (Choose 3)

1 Worry about medical errors 2 Worry about patient complaints 3 Low income and benefits 4 Overtime work, night shifts

5 Bleak prospects 6 Health damage 7 Tense colleague relationships 8 Heavy workload

9 Lack of knowledge and skills 10 Other (note: ________)

2.7 Your marital status: 1 Unmarried 2 Married 3 Divorced 4 Other

2.7.1 If 1 (Unmarried), do you find it difficult to find a partner?

1 Very easy 2 Easy 3 General 4 Not easy 5 Very difficult

2.7.2 If 1 (Unmarried), what are the main factors limiting you from finding an ideal partner? (Choose 3)

1 Work intensity 2 Work nature 3 Interests and hobbies 4 Social circles 5 Family circumstances

6 Length of education 7 Income level 8 Other (note: ______)

2.7.3 If 2 (Married), your spouse's occupation: 1 Doctor 2 Nurse 3 Other medical personnel 4 Non-medical personnel

2.7.4 If 2 (Married), how satisfied are you with your family life?

1 Very dissatisfied 2 Dissatisfied 3 General 4 Satisfied 5 Very satisfied

2.7.5 If 2 (Married), what are the main factors affecting your family life? (Choose 3)

1 Nature and intensity of work 2 Leisure interests 3 Spousal communication 4 Compatibility of personalities 5 Sex life

6 Relationships between family members 7 Elderly and children issues 8 Financial arrangements 9 Other (note): ______

2.8 In the past month, how often did you experience symptoms like "physical fatigue, discomfort", etc:

1 Almost never 2 Rarely 3 Often 4 Almost always

2.9 In the past month, how often did you experience symptoms like "tension, nervousness, restlessness or irritability":

1 Almost never 2 Rarely 3 Often 4 Almost always

2.10 In the past month, how often did you experience symptoms like "lack of energy, difficulty making decisions or need to recheck things repeatedly":

1 Almost never 2 Rarely 3 Often 4 Almost always

2.11 In the past month, how often did you experience symptoms like "gloom, loss of interest, pessimism or easy crying":

1 Almost never 2 Rarely 3 Often 4 Almost always

III. Job Satisfaction and Career Development

3.1 How satisfied are you with your current job position overall:

1 Very dissatisfied 2 Dissatisfied 3 General 4 Satisfied 5 Very satisfied

3.2 How do you feel about the relationship between your remuneration (including salary and bonuses) and your work contribution:

1 Contribution > Income 2 Income = Contribution 3 Contribution < Income

3.3 How fair do you think the promotion of professional titles is: 1 Unfair 2 No opinion 3 Fair

3.4 If you had the opportunity to choose your career again, would you still choose your current profession? 1 No 2 Yes 3 Unsure

3.5 Do you hope your children will study medicine: 1 No 2 Yes 3 Unsure

3.6 Your evaluation of your current profession is: 1 Sacred 2 Valuable 3 Livelihood 4 Low profession

3.7 The protection of your legitimate rights and interests in practice is: 1 Poor 2 General 3 Good

3.8 Does the hospital provide you with convenience for continuing education: 1 Not provided 2 Provided

3.9 What is your top priority for career development in the next few years? (Choose 3)

1 No specific plan 2 Obtain professional certification 3 Further studies or degree 4 Participate in scientific research

5 Improve operational skills 6 Improve humanities literacy 7 Other (note: _______)

3.10 Is the phenomenon of "needed people unable to enter, redundant people unable to leave" serious in this department: 1 Yes 2 No

3.11 Should the state gradually abolish the personnel establishment of public hospitals: 1 Yes 2 No 3 Hard to say

3.12 Which of the following aspects of professional literacy training do you think young doctors in this hospital should strengthen the most? (Choose 3)

1 Medical knowledge 2 Clinical skills 3 Communication skills 4 Professional values, attitudes, behaviors and ethics

5 Public health and health systems 6 Information management 7 Critical thinking and research

IV. Doctor-Patient Relationship and Professional Ethics

4.1 You feel the current doctor-patient relationship is: 1 Very tense 2 Tense 3 General 4 Harmonious 5 Very harmonious

4.2 How much do patients trust you: 1 Very distrustful 2 Distrustful 3 General 4 Very trusting 5 Trusting

4.3 Last year, how many times did you suffer "verbal abuse" from patients: 1 0 2 1-2 3 3-4 4 >4

4.4 Last year, how many times did you have "physical conflicts" with patients: 1 0 2 1-2 3 >3

4.5 You believe the prominent manifestations of doctor-patient relationship tensions caused by doctors are: (Multiple choice)

1 Poor doctor-patient communication 2 Over-prescription or over-examination 3 Missed diagnosis, misdiagnosis 4 Limitations of medicine

5 Poor service attitude 6 Heavy work pressure 7 Other (note: _______)

4.6 From the doctor's perspective, what are the main causes of poor doctor-patient communication? (Choose 3)

1 Limitations of specialties 2 Emergencies or complex and changing conditions 3 Uncertain efficacy 4 Lack of communication awareness

5 Lack of communication skills 6 Insufficient communication time 7 Overly defensive mentality towards patients 8 Other (note: )

4.7 If a patient insists on having a medically unnecessary MRI, will the doctor write an exam order?

1 Yes 2 Yes, but I would tell him/her I don't want to do this 3 No

4.8 When there is a conflict of interest between doctor and patient, whose interest is usually put first:

1 Patient's interest 2 Hospital's interest 3 Personal interest

4.9 Imagine a critically ill patient urgently needing surgery, but the family is fully informed yet still refuses to sign the consent form. What should the attending doctor do first?

1 Operate immediately 2 Give up surgery, take conservative treatment 3 Prepare for surgery and wait for instructions 4 Other (note)

4.10 What do you think is the best way to properly resolve doctor-patient disputes?

1 Doctor-patient negotiation 2 Litigation 3 Third party mediation 4 Administrative mediation 5 Other (note: _____)

4.11 Do your colleagues around you engage in the following academic misconduct? (Multiple choice)

1 Fabrication 2 Falsification 3 Plagiarism 4 Fabrication of peer review opinions 5 None

4.12 What do you think is the fundamental reason for the repeated prohibition of medical misconduct in China? (Choose 3)

1 Lack of integrity awareness 2 Light punishment 3 Lax journal review 4 Biased evaluation system

5 Inducement of intermediary agencies 6 SCI paper incentives 7 Hospital responsibility not implemented 8 Other (note: ____)

4.13 Is "clinical competence" given priority consideration in the professional title evaluation at this hospital? 1 Yes 2 No

4.14 In which aspects do you think the cultural development of this hospital is relatively good? (Multiple choice)

1 Spiritual culture 2 Behavioral culture 3 Institutional culture 4 Material culture 5 None of the above

4.15 What are the current prominent issues in your hospital's cultural development? (Choose 2)

1 Unclear value orientation 2 Becoming a formality 3 Insufficient funding 4 Leadership does not value it 5 Other (note: ____)

V. New Medical Reforms and Practice Environment

5.1 The overall status of China's current medical practice environment is: 1 Very poor 2 Poor 3 General 4 Good 5 Very good

5.2 The extent to which media public opinion negatively impacts the image of medical staff:

1 None 2 Occasionally 3 Sometimes 4 Always

5.3 The extent to which mass media is biased towards patients when reporting medical disputes:

1 None 2 Occasionally 3 Sometimes 4 Always

5.4 How do you feel about the implementation of the goals to "ensure basic healthcare, strengthen grassroots healthcare, and build mechanisms" in the new round of deepening medical reforms:

1 Not achieved 2 Partially achieved 3 Achieved 4 Hard to say

5.5 Are you satisfied with the effects of the public hospital reforms currently implemented in China?

1 Very dissatisfied 2 Dissatisfied 3 General 4 Satisfied 5 Very satisfied

5.6 What do you think are the main difficulties your hospital encounters in implementing the tiered healthcare system? (Choose 3)

1 Concern about loss of patients 2 Lack of patient cooperation 3 Poor referral channels between tiers 4 Difficulty distributing interests between institutions 5 Lack of medical insurance policy incentives 6 Insufficient primary care capabilities 7 Inadequate supervision 8 Other (note______)

5.7 What do you think are the prominent factors limiting multi-site practice of doctors at your hospital? (Choose 3)

1 Unclear distribution of responsibilities, rights and interests between institutions 2 Lack of hospital support 3 Inconsistent clinical scope with registered practice scope

4 Potential risks to quality and safety 5 Difficulty providing continuous services 6 Heavy workload 7 Other (note: )

5.8 Your attitude towards your hospital participating in building regional healthcare consortia is: 1 Oppose 2 Partially agree 3 Largely agree

5.9 Which of the following goals do you think your hospital is most likely to achieve through participating in regional healthcare consortia? (Choose 3)

1 Curb expansion of large hospitals 2 Promote two-way referral 3 Provide cooperative division of labor and continuity of care 4 Promote sharing of medical resources 5 Improve efficiency of resource utilization 6 Promote rational flow of talents 7 Prioritize prevention and integrate medical services and healthcare 8 Other (note_)

5.10 In building healthcare consortia, government responsibilities should focus on playing a role in: (Multiple choice)

1 Establishing building standards 2 Formulating operating norms 3 Improving assessment mechanisms 4 Promoting linkage of healthcare, insurance and pharmaceuticals

5 Increasing fiscal input 6 Strengthening IT infrastructure 7 Other (note________)

5.11 Which healthcare and medical topics are you most concerned with currently? (Choose 3)

1 Healthy China 2 Social medical services 3 Tiered healthcare 4 Healthcare consortia 5 Medical service fees

6 Multi-site practice 7 Zero drug markup 8 Healthcare-insurance-pharmaceutical trinity 9 Other (note: ________)

5.12 Do you have any suggestions for improving the working conditions and environment for hospital staff:

_______________________________________________________________________________________________________________________________________________________________________________________________________________________________________________.

Thank you for your participation!
