# Supplementary material for: Trends in the practice environment of Chinese healthcare professionals from 2008 to 2023: an age period cohort analysis
Source: Hum Resour Health. 2024 Nov 13;22:76. doi: 10.1186/s12960-024-00954-5 (PMC11562610; doi:10.1186/s12960-024-00954-5)
Supplement: Supplementary file 4 — Supplementary material 4. [file 12960_2024_954_MOESM4_ESM.docx]

**Survey on the Practice Status of Healthcare Personnel**

On behalf of the Strategic Development Department of the Chinese Association for Science and Technology, the Chinese Academy of Medical Sciences/Peking Union Medical College is leading this questionnaire survey of healthcare personnel in 40 hospitals across 8 provinces, in order to understand the practice status and attitudes of healthcare personnel in major hospitals and provide policy information to relevant national departments. The survey adopts the principles of anonymity and voluntariness, and will not collect or associate personal information. It takes about 8 minutes to complete, please answer truthfully. Thank you very much for your contribution to this survey!

April 6, 2023

I. Personal Information

1.1 Your gender:

A) Male

B) Female

1.2 Your age:

A) <25

B) 25-34

C) 35-44

D) 45-54

E) >54

1.3 Your highest education level:

A) Associate degree or below

B) Bachelor's degree

C) Master's degree

D) Doctoral degree

1.4 Your technical title:

A) Junior

B) Intermediate

C) Associate senior

D) Senior

E) Unrated

1.5 Your technical position:

A) Physician

B) Nurse

C) Medical technician/Pharmacist

D) Administrator

1.6 Your average monthly income (RMB):

A) <4000

B) 4001-8000

C) 8001-12000

D) 12001-16000

E) >16000

1.7 The type of your hospital:

A) Provincial comprehensive/specialized hospital

B) Municipal comprehensive hospital

C) County comprehensive hospital

D) Traditional Chinese medicine hospital

E) Private hospital

1.8 The level of your hospital:

A) Grade 3A

B) Grade 3B

C) Grade 2A

D) Grade 2B

E) Unrated

1.9 Your department:

A) Internal medicine

B) Surgery

C) Obstetrics and gynecology

D) Pediatrics

E) Emergency

F) Other clinical departments

G) Medical technology/Pharmacy

H) Administration

1.10 Your employment status:

A) On duty

B) Not on duty

1.11 Your current marital status:

A) Unmarried, no stable partner

B) Unmarried, with stable partner

C) Married

D) Divorced

E) Widowed

1.12 Your province:

A) Beijing

B) Henan

C) Shaanxi

D) Zhejiang

E) Guangdong

F) Jiangsu

G) Chongqing

H) Sichuan

I) Xinjiang

II. Work Life and Physical and Mental Health

2.1 Your average daily working hours:

A) <8 hours

B) 8-9 hours

C) 9-10 hours

D) ≥10 hours

2.2 The number of night shifts you work per month:

A) 0

B) 1-4

C) 4-8

D) ≥9

2.3 In addition to your routine medical work, are you engaged in any of the following work? (Multiple choice)

A) Scientific research [Skip to 2.3.1]

B) Teaching

C) Management

D) Health education/Popular science

E) Others _________________

F) None

2.3.1 What are the prominent problems you encounter when conducting scientific research? (Choose 3 options)

A) Busy with clinical work

B) Disconnection between scientific research and clinical practice

C) Lack of research team

D) Lack of research skills

E) Lack of research interest

F) Lack of research funding

G) Cumbersome reimbursement procedures

H) Others _________________

2.4 How much work pressure do you feel:

A) Low

B) Relatively low

C) Moderate

D) Relatively high

E) High

2.5 What are your main sources of pressure at work currently? (Multiple choice)

A) Worry about medical errors

B) Worry about patient complaints

C) Tense interpersonal relationships

D) Lack of knowledge and skills

E) Heavy workload

F) Overtime work, night shifts

G) Low income and benefits

H) Health impairment

I) Bleak prospects

J) Others _________________

2.6 In the past month, how often did you experience symptoms like "physical fatigue, discomfort", etc.?

A) Almost never

B) Occasionally

C) Often

D) Almost always

2.7 In the past month, how often did you experience symptoms like "tension, nervousness, restlessness or irritability"?

A) Almost never

B) Occasionally

C) Often

D) Almost always

2.8 In the past month, how often did you experience symptoms like "listlessness, difficulty making decisions or needing to double-check things"?

A) Almost never

B) Occasionally

C) Often

D) Almost always

2.9 In the past month, how often did you experience symptoms like "gloom, loss of interest, pessimism or easy crying"?

A) Almost never

B) Occasionally

C) Often

D) Almost always

III. Job Satisfaction and Career Development

3.1 How satisfied are you with your current position overall?

A) Dissatisfied

B) Relatively dissatisfied

C) Average

D) Relatively satisfied

E) Satisfied

3.2 What is the relationship between your salary (including wages and bonuses) and your work?

A) Input > Income

B) Income = Input

C) Input < Income

3.3 Do you agree that the salary system in this hospital has achieved "responsibilities and salaries matched to positions, salaries matched to responsibilities, and performance reviews matched to rewards"?

A) Disagree

B) Relatively disagree

C) Average

D) Relatively agree

E) Agree

3.4 How do you feel about the current fairness of promotion in technical titles?

A) Unfair

B) Relatively unfair

C) Average

D) Relatively fair

E) Fair

3.5 Which of the following factors play a key role in the evaluation and promotion of technical titles in your opinion? (Multiple choice)

A) Professional ethics

B) Professional practice skills

C) Scientific research projects/papers

D) Workload and performance

E) Interpersonal relationships

F) Administrative positions

G) Others _________________

3.6 Do you agree that the technical title evaluation for health technicians should implement a results portfolio system (such as papers, reports, medical records, surgical videos, etc.)?

A) Disagree

B) Relatively disagree

C) Average

D) Relatively agree

E) Agree

3.7 If you had the opportunity to choose your career again, would you still choose your current profession?

A) No

B) Yes

C) Hard to say

3.8 Do you want your children to study medicine?

A) No

B) Yes

C) Hard to say

3.9 What is your top priority for career development in the next few years? (Multiple choice)

A) No specific plans

B) Obtain professional certificates

C) Further education or degrees

D) Participate in scientific research

E) Improve operational skills

F) Improve cultural literacy

G) Others _________________

3.10 Which of the following professional qualities do you most need to strengthen? (Multiple choice)

A) Basic medical knowledge

B) Clinical skills

C) Communication skills

D) Professional values, attitudes, behaviors and ethics

E) Public health and health systems

F) Information management

G) Critical thinking

H) Others _________________

3.11 To what extent has this hospital provided you with opportunities and platforms for career development to give full play to your talents?

A) Relatively small

B) Average

C) Relatively large

3.12 In your opinion, what aspects should China improve in terms of the standardized residency training system? (Multiple choice)

A) Improve incentive mechanisms for clinical teaching

B) Guarantee residents' benefits

C) Strengthen training process management

D) Strict training assessments

E) Strengthen humanities education

F) Strengthen the faculty team building

G) Others _________________

IV. Doctor-Patient Relationship and Professional Ethics

4.1 How do you feel about the current doctor-patient relationship?

A) Tense

B) Somewhat tense

C) Average

D) Relatively harmonious

E) Harmonious

4.2 To what extent do patients trust you?

A) Distrust

B) Relatively distrust

C) Average

D) Relatively trust

E) Trust

4.3 Last year, how many times did you suffer "verbal abuse" from patients?

A) 0

B) 1-2 times

C) 3-4 times

D) >4 times

4.4 Last year, how many times did patients have "physical conflicts" with you?

A) 0

B) 1-2 times

C) >2 times

4.5 In your opinion, the tension in doctor-patient relationships due to medical reasons is prominently manifested in: (Multiple choice)

A) Poor doctor-patient communication

B) Over-prescription or over-examination

C) Missed diagnosis or misdiagnosis

D) Limitations of medicine

E) Poor service attitude

F) Heavy work pressure

G) Others _________________

4.6 When you believe a terminally ill patient still has a glimmer of hope for survival, but the patient's guardian clearly requests to withdraw life support. Will you agree to the guardian's request?

A) No

B) Yes

C) It depends

4.7 When the patient's guardian requests life-sustaining treatment that you believe is futile. Will you agree to such a request?

A) No

B) Yes

C) It depends

4.8 In the event of a sudden public health incident, will you obey dispatch to participate in emergency response and medical treatment?

A) No

B) Yes

C) It depends

V. Practice Environment

5.1 How do you feel about the overall status of the medical practice environment in China now?

A) Poor

B) Relatively poor

C) Average

D) Relatively good

E) Good

5.2 How do you feel about the cultural development in your hospital? [Matrix single choice]

|  | Poor | Relatively poor | Average | Relatively good | Good |
| --- | --- | --- | --- | --- | --- |
| Spiritual culture (philosophy, concept, public welfare, etc.) | ○ | ○ | ○ | ○ | ○ |
| Institutional culture (rules, technical norms, etc.) | ○ | ○ | ○ | ○ | ○ |
| Behavioral culture (service level, words and deeds, etc.) | ○ | ○ | ○ | ○ | ○ |
| Material culture (medical conditions, visit environment, etc.) | ○ | ○ | ○ | ○ | ○ |

5.3 What are the prominent problems in the cultural development of your hospital? (Multiple choice)

A) Ambiguous value guidance

B) Just going through the motions

C) Little capital investment

D) Lack of leadership attention

E) Others _________________

5.3.1 What kind of meaningful cultural development do you think your hospital should focus on? (Multiple choice)

A) Strengthen cultural infrastructure

B) Organize thematic activities

C) Improve employee rights protection measures

D) Carry out free clinic or health education

E) Establish a professional honor system

F) Others _________________

5.3.2 If your hospital increases investment in cultural development, which of the following aspects do you think it should be used for? (Multiple choice)

A) Improve working conditions

B) Improve employee benefits

C) Medical assistance for patients in need

D) Organize hospital history and culture

E) Financial assistance for employees in need

F) Others _________________

5.4 Do you agree that the mainstream media is now more shaping the positive image of healthcare workers?

A) Disagree

B) Relatively disagree

C) Average

D) Relatively agree

E) Agree

5.5 Do you agree that mainstream media often favors patients when reporting medical disputes?

A) Disagree

B) Relatively disagree

C) Average

D) Relatively agree

E) Agree

5.6 Overall, how satisfied are you with China's COVID-19 prevention and control measures?

A) Dissatisfied

B) Relatively dissatisfied

C) Average

D) Relatively satisfied

E) Satisfied

5.7 In your opinion, what significant progress has your hospital made in improving medical service quality in recent three years? (Multiple choice)

A) Ensure medical service safety

B) Improve medical technology

C) Promote service continuity

D) Improve service convenience

E) Enhance service comfort

F) Others: _________________

G) Hard to say

5.8 In your opinion, which of the following aspects should the country focus on to improve the medical and health service system? (Multiple choice)

A) Stable public health investment mechanism

B) Medical service pricing mechanism reflecting technology labor value

C) Establish personnel establishment standards and additional mechanisms for public hospitals

D) Deepen salary system reform

E) Give full play to IT support

F) Improve diversified and comprehensive supervision system

G) Others _________________

H) Hard to say

5.9 Do you have any suggestions for improving the working conditions and environment for healthcare workers in China? [Open question]
